# Telmisartan Ameliorates Fibrocystic Liver Disease in an Orthologous Rat Model of Human Autosomal Recessive Polycystic Kidney Disease

**DOI:** 10.1371/journal.pone.0081480

**Published:** 2013-12-06

**Authors:** Daisuke Yoshihara, Masanori Kugita, Mai Sasaki, Shigeo Horie, Koichi Nakanishi, Takaaki Abe, Harold M. Aukema, Tamio Yamaguchi, Shizuko Nagao

**Affiliations:** 1 Education and Research Center of Animal Models for Human Diseases, Fujita Health University, Toyoake, Aichi, Japan; 2 Department of Urology, Juntendo University, Graduate School of Medicine, Bunkyou, Tokyo, Japan; 3 Department of Pediatrics, Wakayama Medical University, Wakayama City, Wakayama, Japan; 4 Division of Medical Science, Tohoku University Graduate School of Biomedical Engineering, Sendai, Miyagi, Japan; 5 Department of Human Nutritional Sciences, University of Manitoba, Winnipeg, Manitoba, Canada; University of Catania, Italy

## Abstract

Human autosomal recessive polycystic kidney disease (ARPKD) produces kidneys which are massively enlarged due to multiple cysts, hypertension, and congenital hepatic fibrosis characterized by dilated bile ducts and portal hypertension. The PCK rat is an orthologous model of human ARPKD with numerous fluid-filled cysts caused by stimulated cellular proliferation in the renal tubules and hepatic bile duct epithelia, with interstitial fibrosis developed in the liver. We previously reported that a peroxisome proliferator activated receptor (PPAR)-γ full agonist ameliorated kidney and liver disease in PCK rats. Telmisartan is an angiotensin receptor blocker (ARB) used widely as an antihypertensive drug and shows partial PPAR-γ agonist activity. It also has nephroprotective activity in diabetes and renal injury and prevents the effects of drug-induced hepatotoxicity and hepatic fibrosis. In the present study, we determined whether telmisartan ameliorates progression of polycystic kidney and fibrocystic liver disease in PCK rats. Five male and 5 female PCK and normal control (+/+) rats were orally administered 3 mg/kg telmisartan or vehicle every day from 4 to 20 weeks of age. Treatment with telmisartan decreased blood pressure in both PCK and +/+ rats. Blood levels of aspartate amino transferase, alanine amino transferase and urea nitrogen were unaffected by telmisartan treatment. There was no effect on kidney disease progression, but liver weight relative to body weight, liver cystic area, hepatic fibrosis index, expression levels of Ki67 and TGF-β, and the number of Ki67- and TGF-β-positive interstitial cells in the liver were significantly decreased in telmisartan-treated PCK rats. Therefore, telmisartan ameliorates congenital hepatic fibrosis in ARPKD, possibly through the inhibition of signaling cascades responsible for cellular proliferation and interstitial fibrosis in PCK rats. The present results support the potential therapeutic use of ARBs for the treatment of fibrocystic liver disease in ARPKD patients.

## Introduction

Hereditary polycystic kidney diseases (PKDs) are characterized by progressive enlargement of countless fluid-filled cysts in the bilateral kidneys, and most cases also produce liver cystic disorders. Cyst formation is caused by stimulated proliferation of the renal tubule and hepatic bile duct epithelia, together with secretion of fluid into the cyst cavities. PKD occurs in two major types, autosomal dominant PKD (ADPKD) and autosomal recessive PKD (ARPKD). The incidence of ADPKD in humans is 1∶500–1,000, and ADPKD is caused by mutations in either the *PKD1* or *PKD2* gene [1]. Most cases of ADPKD are diagnosed in adulthood, and end-stage renal disease occurs in 50% of patients [2]. In contrast, ARPKD is a juvenile type cystic disease with an incidence of 1∶20,000 [3] which is caused by the *PKHD1* gene. Renal cysts originate from collecting ducts with fibrotic tissue. Liver cysts originate from intrahepatic bile ducts and are connected to the biliary tree, which appears distorted and embedded in abundant fibrotic tissue [4]. Renal insufficiency and end-stage renal disease are remarkable symptoms in neonatal patients, and complications of ductal plate malformation caused by congenital hepatic fibrosis (CHF) are prominent in adult survivors [4].

Angiotensin II type 1 receptor blockers (ARBs) are widely used as antihypertensive drugs in patients with renal diseases including ARPKD. Hypertension is a critical factor exacerbating ARPKD, and early-onset of severe hypertension frequently occurs in childhood and at young ages [4], [5], [6]. In the liver, CHF is often complicated by portal hypertension [4], [5], [6]. Upregulation of intrarenal renin and angiotensin-converting enzyme is observed in a human-gene orthologous rodent model [7]. One of the ARBs, telmisartan, is known as an agonist of peroxisome proliferator activated receptor (PPAR)-γ, which is a ligand-activated transcription factor belonging to the nuclear hormone receptor superfamily [8]. PPAR-γ agonists regulate signaling pathways related to the inflammatory response, cellular proliferation, and fibrotic changes [9], [10], [11]. Telmisartan has preventive effects against hepatotoxicity and hepatic fibrosis [12], [13] as well as nephron-protective activity in certain models, possibly through the regulation of intracellular signaling events.

At present, there is no established drug treatment for ARPKD patients. We previously reported that pioglitazone, a PPAR-γ full agonist, ameliorates kidney and liver disease progression in PCK rats, an orthologous model of human ARPKD [14], [15], [16]. However, increased risk of bladder cancer is a concern with the long term clinical use of pioglitazone [17], [18], [19]. Therefore, in the present study, we selected telmisartan as it is a partial PPAR-γ agonist, and already is being used for long term treatment in kidney disease patients with hypertension. We determined whether telmisartan ameliorates the three major symptoms of ARPKD, polycystic kidney disease, fibrocystic liver disease, and hypertension by its PPAR-γ agonist and angiotensin II type 1 receptor blockade activity in this orthologous rat model of human ARPKD.

## Materials and Methods

### Animals and Study Design

The PCK rat strain was originally derived from a Sprague-Dawley colony maintained in Japan. Renal and hepatic diseases are caused by a splicing mutation with subsequent skipping of exon 36 and a frameshift in the human orthologous *Pkhd1* gene [20]. Disorders in PCK rats are characterized by renal cysts derived from collecting ducts, and congenital hepatic fibrosis associated with biliary cysts [14], [21], [22]. Descendants from the original colony have been bred and maintained at the Education and Research Center of Animal Models for Human Diseases, Fujita Health University. In the present study, PCK rats and normal Sprague-Dawley (+/+) rats (Charles River Japan Inc., Kanagawa, Japan) were allowed free access to water and food. Male and female PCK and +/+ rats (*n* = 10 per gender) were randomly assigned to one of two groups (*n* = 5 per group): treatment with 3 mg/kg telmisartan (Tokyo Chemical Industry Co., Ltd. Tokyo, Japan) or vehicle (0.3% dimethyl sulfoxide, Sigma, St. Louis, MO, USA) by gavage every day from 4 to 20 weeks of age.

Systolic blood pressure (SBP) was measured in 4- and 19-week-old conscious rats by tail-cuff sphygmomanometer (BP98A, Softron Co. Ltd. Tokyo, Japan). At 20 weeks of age, rats were anesthetized with sodium pentobarbital (Schering-Plough Corp., Kenilworth, NJ), and the kidneys and liver were removed rapidly, causing lethal exsanguination. Body weight, total kidney weight, and liver weight were measured, and blood samples were collected for measurements of serum urea nitrogen (SUN), a marker of kidney function. Serum aspartate amino transferase (AST) and alanine aminotransferase (ALT), were measured as markers of liver function.

Half of the left kidney and 0.5 g of the liver were homogenized in ice-cold triton lysis buffer to extract proteins [14], [21]. Half of the right kidney and a part of the right medial liver lobe were embedded in 4% carboxy methyl cellulose (FINETEC Co., Ltd, Tokyo, Japan) after fixation in 4% paraformaldehyde (PFA) for 60 min at 4°C and sequential incubations in 10, 20, and 30% sucrose for 30, 60, and 120 min, respectively at 4°C and were sectioned for immunofluorescence staining. The other half of the right kidney and a part of the right medial liver lobe were immersed in 4% PFA overnight at 4°C, displaced in phosphate buffered saline without Mg^2+^ and Ca^2+^ (PBS^−^), dehydrated by ethanol, dealcoholized by xylene, embedded in paraffin, and sectioned for immunohistochemistry.

### Ethics Statement

Animals were handled according to the Regulations for the Management of Laboratory Animals at Fujita Health University. The protocol for the ethical use of these animals was approved by the Animal Care and Use Committee at Fujita Health University (Permit No.: M2806).

### Measurement of SUN, AST, ALT and Angiotensin II Levels

SUN measurements were performed with a colorimetric assay using a urease-indophenol method (Wako Pure Chemicals, Osaka, Japan). AST and ALT were determined by the pyruvate oxidase-N-ethyl-N-(2-hydroxy-3-sulfopro-pyl)-m-toluidine method with a commercial kit (Transaminase CII-test; Wako). Serum angiotensin II measurements were performed with enzyme immunoassay (Enabling Discovery in Life Science, Plymouth Meeting, PA, USA). SUN, AST, ALT and angiotensin II levels are expressed as mean±standard error (SE) (n = 5 rats per treatment group for each gender).

### Cyst Index and Fibrosis Index

Cystic area was measured from 5 random fields (×100 magnification) of hematoxylin and eosin-stained kidney and liver sections from PCK and +/+ rats. Fibrosis area was measured from 5 random fields (×100 magnification) of picrosirius red-stained sections of both organs. The picrosirius red-stained sections were also observed for collagen fibers by polarized light microscopy [23]. Cystic and fibrotic indices (%total field) were measured and calculated by a naive observer using LUZEX FS software (Kideko CO.LTD, Tokyo, Japan) and are expressed as mean±SE values.

### Western Blot Analysis

Proteins (20 or 100 µg protein per lane) from kidney or liver tissue lysates were separated by 10% sodium dodecyl sulfate-polyacrylamide gel electrophoresis and were transferred to nitrocellulose membranes. Membranes were blocked with 5% milk in Tris buffer solution-tween20 (TBS-T) for 1 h at room temperature and incubated overnight at 4°C in primary antibody in 2% milk in TBS-T. Primary antibodies were anti-ERK1/2 (SC-94, dilution 1∶1,000; Santa Cruz Biotechnology, Santa Cruz, CA, USA), anti-pERK1/2 (SC-7383, dilution 1∶1,000; Santa Cruz), anti-TGF-β (#3711, dilution 1∶1,000, Cell Signaling Technology, Danvers, MA, USA), anti-angiotensin II type 1 receptor (AT1R, ab18801, dilution 1∶1000, Abcam, Cambridge, UK), and anti-GAPDH (ab8245, dilution 1∶10,000, Abcam). Membranes were then washed three times with TBS-T and incubated with secondary antibody (Santa Cruz) conjugated to horseradish peroxidase in 2% milk in TBS-T. Specific antibody signals were detected with an enhanced chemiluminescence system (ECL or ECL Advance Western Blotting Detection System; Amersham Life Sciences, Arlington Heights, IL, USA). Images of the blots were captured, and the intensity of the protein bands was quantified by a CS Analyzer 2.0 with a CCD camera (ATTO Corporation, Tokyo, Japan). The relative band intensity from the PCK rats was compared with that of gender- and age-matched +/+ kidneys (set to 100%).

### Immunohistochemistry and Immunofluorescence

Paraffin sections were deparaffinized by xylene, rehydrated to PBS^−^ from ethanol, then heated in a microwave to 100°C for 15 min in 10 mM sodium citrate buffer (pH 6.0) to unmask antigens. Endogenous peroxidase was destroyed by incubating sections in 0.3% H_2_O_2_/methanol. The sections were blocked with 1% bovine serum albumin and 0.05% NaN_3_ in PBS^−^ for 30 min at room temperature and were incubated with primary antibody overnight at 4°C. Primary antibodies for immunohistochemistry were anti-ERK1/2 (SC-94, dilution 1∶200; Santa Cruz), anti-pERK1/2 (M8159, dilution 1∶200; Sigma), anti-Ki67 (ab16667, dilution 1∶100; Abcam), anti-ERK5 (SC-1284, dilution 1∶100; Santa Cruz), and anti-pERK5 (#3371, dilution 1∶50; Cell Signaling). Rinsed sections were incubated with biotinylated anti-mouse and anti-rabbit IgG/IgA/IgM secondary antibody (Histofine, Nichirei Biosciences, Tokyo, Japan) for immunohistochemical staining. Immune reaction products were developed with 3,3′-diaminobenzidine. For immunofluorescence detection, the 4% PFA-fixed thin sections were incubated with a primary antibody against TGF-β (SC-146, dilution 1∶100; Santa Cruz) overnight at 4°C. Rinsed sections were incubated with anti-mouse IgG secondary antibody conjugated to Alexa568 (Invitrogen Corporation, Carlsbad, CA, USA). Positively-stained cells were counted in five random fields of kidney and liver sections obtained from five rats in each group by a naive observer using a 40× objective.

### Statistics

Results are expressed as mean ± SE values. Statistical comparisons were made with the Student *t*-test or two-way analysis of variance, as appropriate, and differences were considered significant at *P*<0.05.

## Results

### Effect of Telmisartan on Blood Pressure

**Figure 1:**
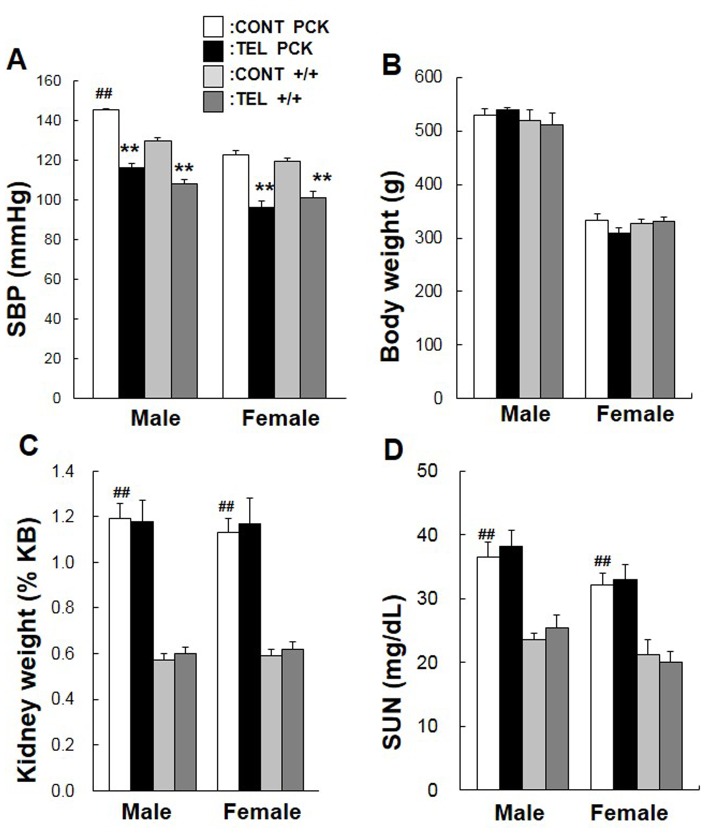
Effect of telmisartan on blood pressure, kidney weight, and renal function. Male and female PCK and +/+ rats (n = 5 per group) received 3 mg/kg telmisartan (TEL) or control vehicle (0.3% dimethyl sulfoxide, CONT) by gavage every day from 4 to 20 weeks of age. Blood pressure (A) is represented by systolic blood pressure (SBP). Kidney weights (%KB, C) are presented as a percent of total body weight (B). Serum urea nitrogen (SUN) was measured as a renal function marker (D). Comparison between control vehicle-treated (CONT) PCK and +/+ rats (female or male), ^##^ *P*<0.01. Comparison between vehicle-treated (CONT) and telmisartan-treated (TEL) PCK and +/+ rats (female or male), **P<0.01.

SBP was significantly higher in PCK male rats compared with +/+ male rats at 19 weeks of age (*P*<0.01) (Figure 1A), whereas no difference was seen at 4 weeks of age (data not shown). On the other hand, in female PCK rats, SBP was not different from that in the +/+ rats at 4 (data not shown) or 19 weeks of age (Figure 1A). At 19 weeks of age, telmisartan decreased SBP by 20% (*P*<0.01) in male and by 22% (*P*<0.01) in female PCK rats, and by 17% (*P*<0.01) in male and by 15% (*P*<0.01) in female +/+ rats (Figure 1A).

### Effect of Telmisartan on Kidney Weight and Renal Function

Body weight did not differ between gender-matched PCK and +/+ rats at 20 weeks of age and was unaffected by the treatment with telmisartan (Figure 1B). Percent kidney weight relative to body weight was 2.1- and 1.9-fold greater in PCK male (*P*<0.01) and female (*P*<0.01) rats compared with gender-matched +/+ rats (Figure 1C). SUN levels in both genders of PCK rat were higher compared with gender-matched +/+ rats (*P*<0.01, Figure 1D), even though the levels in PCK rats were not indicative of renal insufficiency. Percent kidney weight relative to body weight and SUN levels were unaffected by the treatment with telmisartan.

### Effects of Telmisartan on Hepatic Cysts and Liver Function

**Figure 2:**
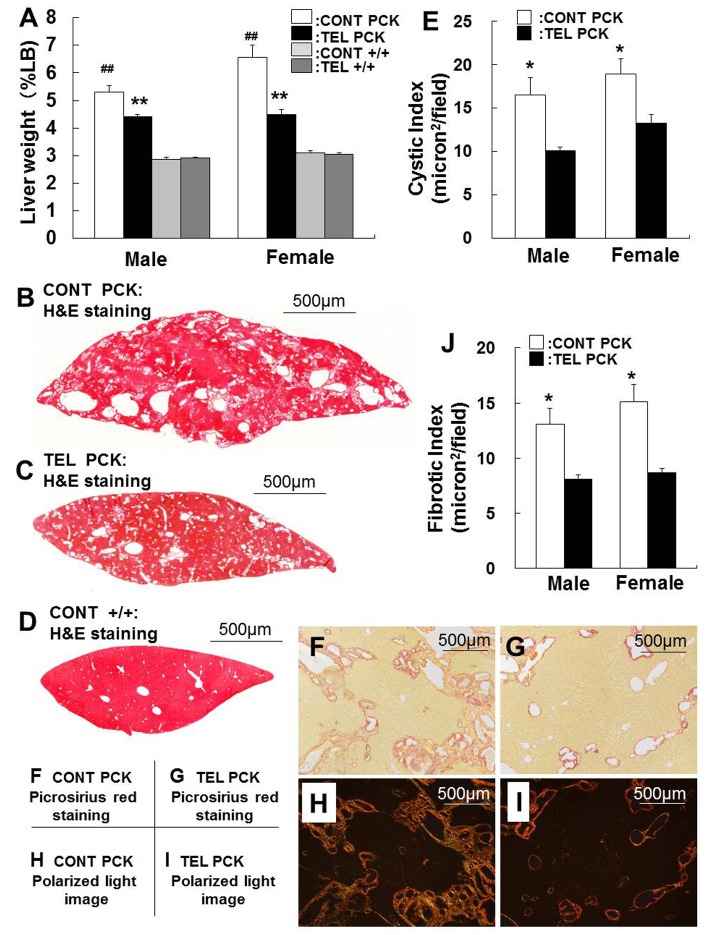
Effect of telmisartan on liver weight, hepatic cysts, and hepatic fibrosis. Liver weights (%LB, A) are presented as a percent of total body weight. Representative liver sections from male rats were stained with hematoxylin and eosin (H&E) [B: control vehicle-treated (CONT) PCK liver, C: telmisartan-treated (TEL) PCK liver, D: control vehicle-treated (CONT) +/+ liver]. Cystic index (E) was determined from representative liver sections by morphometric analysis. Pathogenic fibrosis is indicated by picrosirius red staining in optical [F: control vehicle-treated (CONT), G: telmisartan-treated (TEL)] and in circularly polarized light images [H: control vehicle-treated (CONT), I: telmisartan-treated (TEL)]. Fibrotic index (J) was determined from representative liver sections by morphometric analysis. Comparison between vehicle-treated (CONT) PCK and +/+ rats (female or male), ^##^ *P*<0.01. Comparison between vehicle-treated (CONT) and telmisartan-treated (TEL) PCK and +/+ rats (female or male), *P<0.05, **P<0.01.

Liver weight relative to body weight was 1.8- and 2.1-fold greater in male (*P*<0.01) and female (*P*<0.01) PCK rats, respectively, compared with gender-matched +/+ rats (Figure 2A). Telmisartan decreased liver weight by 16% in male (P<0.01) and 31% in female (P<0.01) PCK rats, whereas liver weight of +/+ rats in both genders was unaffected by the treatment with telmisartan (Figure 2A).

In the liver sections from telmisartan-treated PCK rats, the cysts were smaller and the obvious cysts were fewer than those in vehicle-treated specimens (Figure 2B and C, The vehicle-treated +/+ liver is shown in Figure 2D). The decreased cyst area both in male (*P*<0.05) and female (*P*<0.05) PCK rats is shown in Figure 2E. Serum AST levels were higher in PCK rats compared with +/+ rats both in vehicle-treated (P<0.01) and telmisartan-treated (P<0.05) females but not in males (Figure S1A). ALT levels were in the normal range in both genders of PCK rats (Figure S1B). Neither AST nor ALT were affected by telmisartan treatment (Figure S1A and B).

### Effects of Telmisartan on Hepatic Fibrosis

Picrosirius red staining was performed for the morphological evaluation of fibrosis. Optical microscopic (Figure 2F and G) and polarized light images (Figure 2H and I) of picrosirius red sections were similar. Telmisartan significantly decreased the fibrotic area per total liver section in male (*P*<0.05) and female (*P*<0.05) PCK rats (Figure 2J).

### Effects of Telmisartan on Hepatic Cell Proliferation

**Figure 3:**
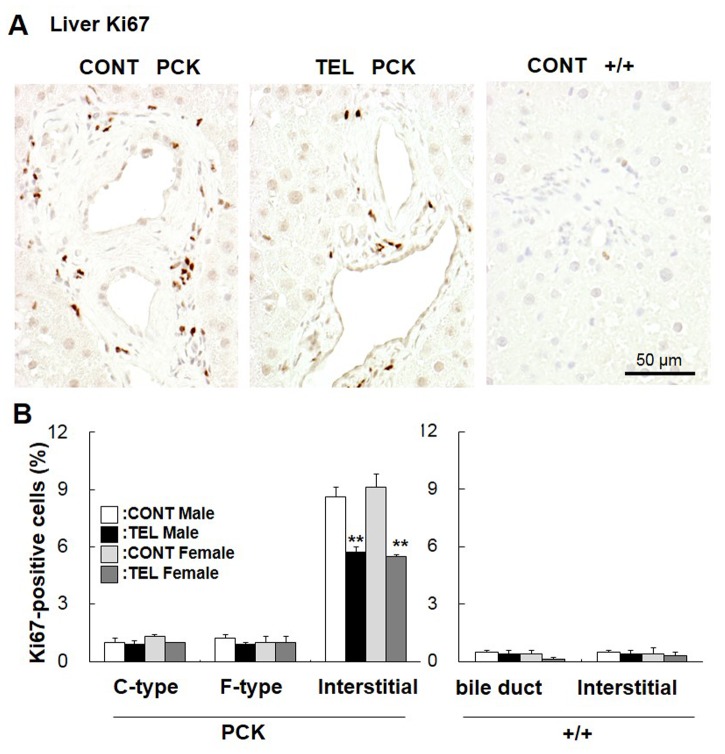
Effect of telmisartan on cell proliferation. Liver sections from PCK and +/+ rats were stained with an antibody to Ki67, a marker of proliferation activity (A). The percentage of Ki67-positive cells (B) was determined in cuboidal-shaped (C-type) and flat-shaped (F-type) cholangiocytes and the interstitial regions of PCK liver sections, and for normal bile ducts and the interstitial cells of +/+ livers. The nucleus of Ki67-positive cells was stained dark brown by the immunohistochemical reaction, and that of Ki67-negative cells appeared blue because of the counterstaining with hematoxylin. Comparison between vehicle-treated (CONT) and telmisartan-treated (TEL) PCK or +/+ rats of each gender, **P<0.01.

In the liver, the total number of Ki67-positive cells was higher in PCK rats than in +/+ rats (Figure 3A). In PCK rats, telmisartan significantly decreased the number of Ki67-positive interstitial cells (*P*<0.01, Figure 3B). In the hepatocytes, no significant difference was seen between samples from telmisartan-treated and vehicle-treated rats (data not shown). In cuboidal-shaped and flat-shaped cholangiocytes, the expression of Ki67 was unaffected; whereas in interstitial cells, it was significantly reduced by telmisartan treatment in both genders (*P*<0.01). In +/+ livers, hepatocytes, epithelial cells of the bile duct, and interstitial cells with positive Ki67 staining was fewer and unaffected by telmisartan treatment (Figure 3A and B).

### Effects of Telmisartan on TGF-β Signaling Pathway in Liver Tissue

**Figure 4:**
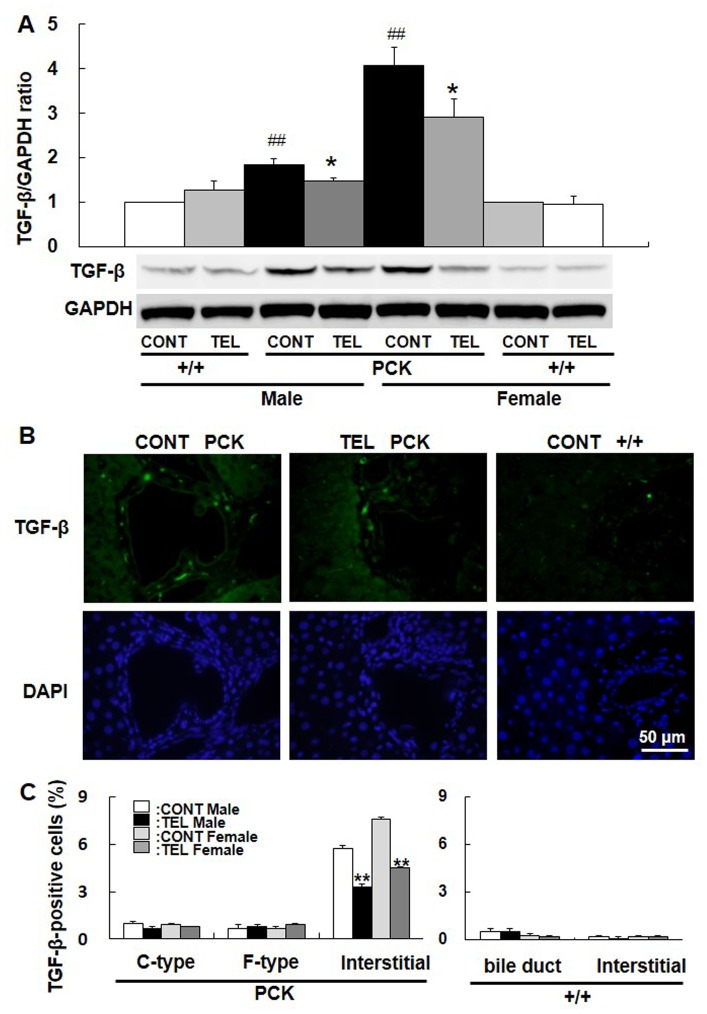
Effect of telmisartan on TGF-β expression. Protein bands were probed with an antibody to TGF-β or GAPDH, and the ratio of TGF-β/GAPDH was determined from blots by density analysis (A). Liver sections from PCK and normal +/+ rats were stained with an antibody to TGF-β. TGF-β-positive cells were stained green by Alexa568, and nuclei were stained blue by DAPI (B). The percentage of TGF-β-positive cells was determined in cuboidal-shaped (C-type) and flat-shaped (F-type) cholangiocytes and the interstitial regions of PCK liver sections, and in normal bile ducts and the interstitial cells of +/+ liver sections (C). Comparison between vehicle-treated (CONT) PCK and +/+ rats in each gender, ^##^ *P*<0.01. Comparison between vehicle-treated (CONT) and telmisartan-treated (TEL) PCK or +/+ rats in each gender, *P<0.05, **P<0.01.

TGF-β is associated with the progression of hepatic fibrosis in PCK rats [14]. To determine the effects of telmisartan, TGF-β expression in liver tissue was measured. We found that the abundance of TGF-β in the tissue lysate was significantly increased in PCK rats compared with +/+ rats (*P*<0.01, Figure 4A), and it was decreased by treatment with telmisartan in both genders (*P*<0.05, Figure 4A). In liver sections, the percentage of TGF-β-positive cells was significantly higher in PCK rats compared with +/+ rats (Figure 4B and C), and it was decreased by treatment with telmisartan in both genders (*P*<0.01, Figure 4C). Inflammation causes activation of hepatocyte stellate cells (HSCs) in the liver. Activated HSCs secrete TGF-β, which promotes epithelial to mesenchymal transition from hepatocytes and cholangiocytes to myofibroblasts to induce interstitial fibrosis [24], [25]. Hence, these results suggest that telmisartan may inhibit fibrosis by suppressing inflammatory activity.

### Effects of Telmisartan on ERK Signaling Pathways in Liver Tissue

To determine the effects of telmisartan on cellular signaling events associated with proliferation, the expression of pERK1/2 and pERK5 in liver specimens was measured. The expression of pERK1/2 and pERK5 was significantly higher in PCK rats compared with +/+ rats (data not shown), and it was unaffected by the treatment with telmisartan.

### Effect of Telmisartan on Angiotensin II and AT1R

To determine the effects of telmisartan on the renin-angiotensin system in the liver, the concentration of serum angiotensin II and expression of AT1R in tissue specimens were measured. Serum angiotensin II was significantly higher in PCK rats compared with +/+ rats at 20 weeks of age (male P<0.05, female P<0.05), and telmisartan treatment increased serum angiotensin II level in both PCK rats (4.67 fold in male P<0.01, 2.13 fold in female P<0.05) and +/+ rats (5.43 fold in male P<0.05, 6.16 fold in female P<0.01) possibly by blocking angiotensin II-induced feedback regulation of renin release [26], [27] (Figure S2A). The expression of AT1R in the liver lysate did not differ between gender-matched PCK and +/+ rats at 20 weeks of age and was unaffected by the treatment with telmisartan (Figure S2B).

## Discussion

ARPKD produces severe clinical manifestations, mostly during the neonatal period, by mutation of *PKHD1* genes on homologous chromosomes; but survival has been increased in juvenile and adult patients by the recent development of medical treatment. ARPKD patients have obviously enlarged kidneys characterized by innumerable fluid-filled cysts. Severe hypertension is shown to be the prominent factor causing deterioration of renal function. Most ARPKD patients have enlarged livers with ductal plate malformation, dilated intrahepatic bile ducts with abnormal branching of the intrahepatic portal veins, and progressive fibrosis of the portal tracts, resulting in CHF/Caroli’s syndrome (CS) and the occurrence of significant portal hypertension [4], [5], [6]. Systemic and portal hypertension is a well-recognized complication of ARPKD. Angiotensin-converting enzyme inhibitors, β-blockers, calcium channel blockers, and diuretics are prescribed as antihypertensive medicines to ARPKD patients; whereas β-blockers are used to treat patients with portal hypertension as well [28], [29]. However, drug-treatment regimens for ARPKD patients have not yet been established.

We previously reported that pioglitazone, a PPAR-γ full agonist, ameliorated kidney and liver disease in PCK rats [14], [15], [16]. PPAR-γ belongs to the superfamily of nuclear hormone receptors and regulates the transcription of a number of target genes controlling essential physiological processes [30]. It is expressed in various tissues including liver [31], and its activity reduces hyperglycemia, dyslipidemia, inflammation, oxidative stress, matrix remodeling, and fibrosis [30], [32]. A decrease in TGF-β signaling activity by PPAR-γ agonists causes the suppression of fibrosis and cell proliferation in the liver [33]. In our previous study, a full PPAR-γ agonist, pioglitazone, was shown to ameliorate hepatic disease progression by suppression of increased TGF-β expression in PCK rats [14]. Another PPAR-γ agonist, rosiglitazone, attenuated the progression of renal disease by inhibiting TGF-β expression in Han:SPRD-Cy rats and inhibited TGF-β-mediated fibrogenes in ADPKD cyst-lining epithelial cells [34], [35]. These findings suggest that PPAR-γ full agonists could be therapeutically effective against cystic disease progression in PKD patients, although higher risk of bladder cancer with the use of pioglitazone or rosiglitazone is a concern [17], [18], [19]. Telmisartan is known to be a partial agonist of PPAR-γ [8], [36]. Furthermore, telmisartan has renoprotective activity brought about through control of blood pressure, decreased vasopressin secretion, and reduced inflammation and cellular proliferation activity [37], [38]. Because telmisartan is already used in kidney disease patients with high blood pressure, its prompt clinical application for ARPKD patients could be feasible. In the present study, telmisartan reduced hypertension and fibrocystic liver disease in PCK rats, although renal disease was not ameliorated. Because high blood pressure is one of the major risks affecting kidney function, it will be intriguing to determine whether treatment with a higher dose [39], [40] or longer period could have renoprotective acted.

In one of our preliminary studies, an antihypertensive drug, hydralazine, a vasodilator without PPAR-γ agonistic action, reduced blood pressure levels, but CHF/CS was not ameliorated (data not shown). In contrast, other ARBs such as olmesartan, losartan, and valsartan are reported to inhibit hepatic fibrosis [41]. In addition, it is reported that hepatic expression of TGF-β is induced by angiotensin II. Administration of angiotensin II causes higher expression of TGF-β in normal rat liver [42] and exacerbates liver fibrosis in bile duct-ligated rats [43]. In the current study, serum angiotensin II concentration was increased in the PCK rats (Figure S2A). Taken together, this suggests that the anti-fibrotic effect in PCK rat livers could be caused, at least in part, by the AT1 receptor blocking activity of telmisartan. In the previous study [14], cystic and fibrotic liver and kidney disease progression was significantly reduced by a full PPAR-γ agonist in PCK rats without angiotensin II receptor blockade, demonstrating that both liver and kidney are sensitive to the beneficial effects of PPAR-γ agonist. But telmisartan only ameliorated liver disease in the present study, suggesting that the partial PPAR-γ agonist activity of telmisartan may not be robust enough to cause the beneficial effect, especially in the kidney. Further, in the present study, TGF-β expression was stimulated in the liver but not in kidney in the current experiment. This may be one of the reasons why no significant effect of telmisartan against kidney disease was shown. If renal expression of TGF-β and occurrence of interstitial fibrosis is increased in the latter stage of disease progression in this model, telmisartan may be effective at ameliorating kidney disease as well.

## Conclusions

The ARB/PPAR-γ partial agonist telmisartan significantly decreases hypertension and fibrocystic liver disease in association with inhibition of the TGF-β signaling pathways in PCK rats, an orthologous model of human ARPKD. Because telmisartan is already a useful medicine for hypertension, it may be an effective therapeutic agent in ARPKD and CHF/CS patients.

## Supporting Information

Figure S1 **Effects of telmisartan on serum AST and ALT.** Serum AST (A) and ALT (B) were measured by the pyruvate oxidase-N-ethyl-N-(2-hydroxy-3-sulfopro-pyl)-m-toluidine method with a commercial kit. Comparison between vehicle-treated (CONT) PCK and +/+ female rats, ^##^P<0.01. Comparison between telmisartan-treated (TEL) PCK and +/+ female rats, $P<0.05.
(TIF)

Figure S2 **Effects of telmisartan on angiotensin II and angiotensin II type 1 receptor in the liver.** Serum angiotensin II (A) was measured by enzyme immunoassay. Comparison between vehicle-treated (CONT) PCK and +/+ rats in each gender, ^#^P<0.05. Comparison between vehicle-treated (CONT) and telmisartan-treated (TEL) PCK or +/+ rats in each gender, *P<0.05, **P<0.01. Protein bands were probed with an antibody to angiotensin II type 1 receptor (AT1R) or GAPDH (B).
(TIF)
