# Supplementary figures and images for: Telmisartan Ameliorates Fibrocystic Liver Disease in an Orthologous Rat Model of Human Autosomal Recessive Polycystic Kidney Disease
Source: PLoS One. 2013 Dec 6;8(12):e81480. doi: 10.1371/journal.pone.0081480 (PMC3855683; doi:10.1371/journal.pone.0081480)

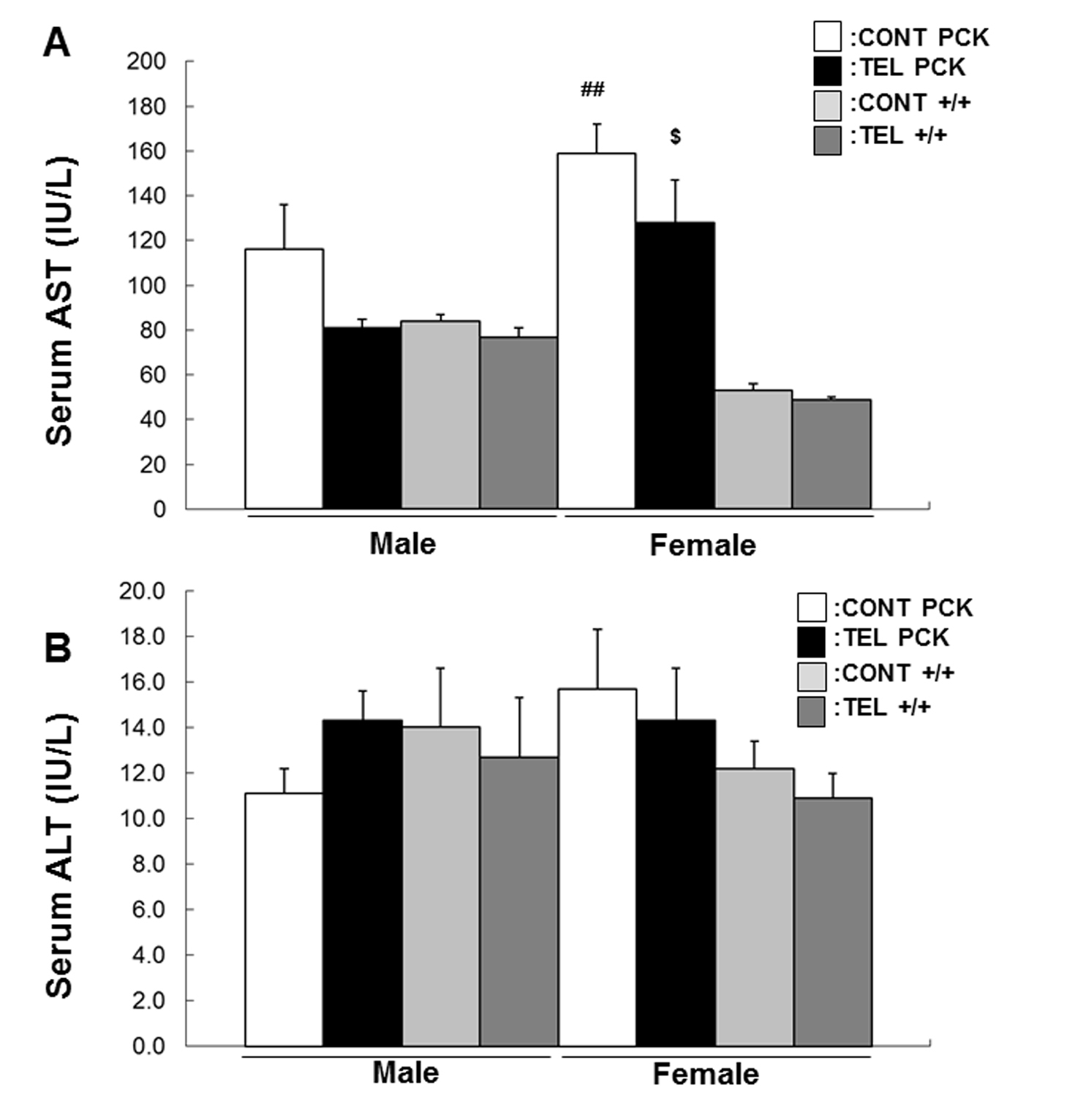

Supplement: Figure S1 — Effects of telmisartan on serum AST and ALT. Serum AST (A) and ALT (B) were measured by the pyruvate oxidase-N-ethyl-N-(2-hydroxy-3-sulfopro-pyl)-m-toluidine method with a commercial kit. Comparison between vehicle-treated (CONT) PCK and +/+ female rats, ##P<0.01. Comparison between telmisartan-treated (TEL) PCK and +/+ female rats, $P<0.05. (TIF) [file pone.0081480.s001.tif]

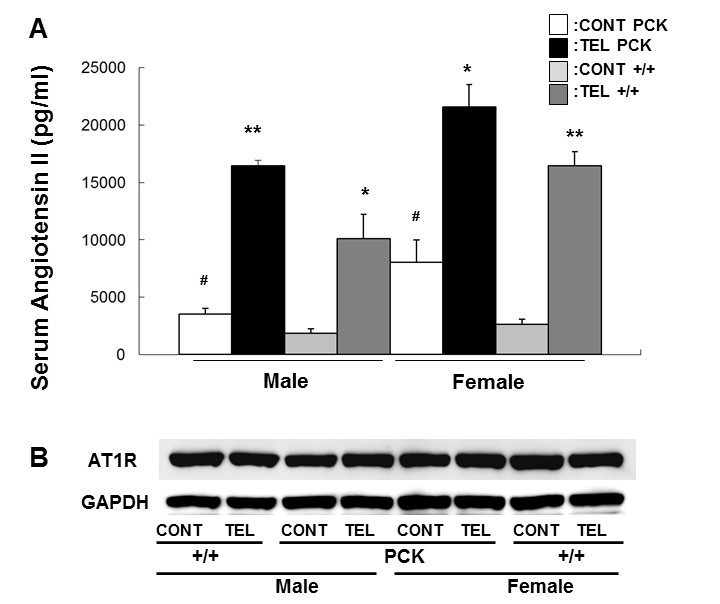

Supplement: Figure S2 — Effects of telmisartan on angiotensin II and angiotensin II type 1 receptor in the liver. Serum angiotensin II (A) was measured by enzyme immunoassay. Comparison between vehicle-treated (CONT) PCK and +/+ rats in each gender, #P<0.05. Comparison between vehicle-treated (CONT) and telmisartan-treated (TEL) PCK or +/+ rats in each gender, *P<0.05, **P<0.01. Protein bands were probed with an antibody to angiotensin II type 1 receptor (AT1R) or GAPDH (B). (TIF) [file pone.0081480.s002.tif]
